# Effect of postmastectomy radiotherapy on triple-negative breast cancer with T1-2 and 1-3 positive axillary lymph nodes: a population-based study using the SEER 18 database

**DOI:** 10.18632/oncotarget.24703

**Published:** 2019-08-27

**Authors:** Jie Zhang, Xiao-Xiao Wang, Jun-Yu Lian, Chuan-Gui Song

**Affiliations:** ^1^ Department of Breast Surgery, Affiliated Union Hospital, Fujian Medical University, Fuzhou, China; ^2^ Department of Gynecology and Obstetrics, Fujian Provincial Maternity and Children Health Hospital, Teaching Hospital of Fujian Medical University, Fuzhou, China

**Keywords:** postmastectomy radiotherapy, triple-negative breast cancer, 1-3 positive lymph nodes, overall survival, breast cancer-specific survival

## Abstract

There is consensus on the routine use of postmastectomy radiotherapy (PMRT) in patients with four or more positive axillary lymph nodes. However, the benefits of PMRT in patients with T1-2 and 1-3 involved lymph nodes still remain controversial. Data from the Surveillance, Epidemiology, and End Results Program (SEER) of the United States between 2010 and 2012 were used to analyze the outcomes of 675 triple-negative breast cancer (TNBC) patients with T1-2 and 1-3 lymph nodes involved. Those patients were subdivided into radiotherapy (RT) (312) and no-RT groups (363). After a median follow-up time of 37 months, Kaplan-Meier analysis showed that PMRT significantly improved overall survival (OS) but not breast cancer-specific survival (BCSS) in the total cohort of 675 patients (*P*=0.033 and *P*=0.063). And it was demonstrated that PMRT were independently associated with increased OS according to univariate and multivariate analyses. However, no significant differences in BCSS or OS were observed between the groups stratified by the number of positive lymph nodes. In conclusion, PMRT significantly improved OS for TNBC patients with T1-2 and 1-3 lymph nodes involved. Additional prospective studies are needed to provide a stronger evidence base for choosing patients for PMRT.

## INTRODUCTION

Breast cancer is a highly heterogeneous disease, which is reflected in different clinical, pathologic, and molecular features. Currently, breast cancers are classified into four molecular subtypes including, luminal A, luminal B, human epidermal growth factor receptor 2 (HER2) overexpressed, and triple-negative breast cancer (TNBC) subtypes according to estrogen receptor (ER), progesterone receptor (PR), and HER2 status by gene expression profiling [1, 2]. The TNBC tumors are defined not having ER, PR, and HER2 expression, and it has a worse prognosis because of its aggressively malignant features compared to the luminal subtypes. Regional recurrence and distance metastasis, especially to visceral organs and soft tissues, occur more frequently in TNBC. In addition, TNBC patients confront a high rate of early recurrence within the first three years and higher mortality within the first five years compared with other subtypes [3, 4].

Adjuvant radiotherapy plays a pivotal role in the treatment of breast cancer, since it can reduce the risk of loco-regional recurrence and overall mortality [5, 6]. And postmastectomy radiotherapy (PMRT) is considered a standard treatment for high-risk breast cancers patients with involvement of more than four positive axillary lymph nodes [7, 8]. However, it still remains controversial for patients with 1-3 lymph nodes to receive radiotherapy (RT). Treatment with PMRT is recommended by the National Comprehensive Cancer Network’s clinical practice guidelines for patients with early-stage breast cancer with 1–3 positive axillary lymph nodes [9]. In the St. Gallen Breast Cancer Meeting in 2017, 54.5% of experts supported PMRT being used as standard treatment for patients with N+ 1 to 3 all patients. A subgroup analysis by the Danish Breast Cancer Cooperative Group (DBCG) 82 B&C randomized trial indicated that the survival benefit after PMRT was substantial and similar in patients with 1–3 and four or more positive lymph nodes [10]. A meta-analysis of 22 randomized trials from Early Breast Cancer Trial list’s Collaboration Group (EBCTCG) in 2014 revealed that PMRT could reduce 10-year loco-regional recurrence and 20-year breast cancer mortality for patients with one to three positive axillary lymph nodes [6]. However, the retrospective multicenter analysis (KROG 1418) reported that PMRT had no significant impact on loco-regional recurrence-free survival (LRRFS), disease-free survival (DFS), and overall survival (OS) in pT1-2N1 patients treated with taxane-based chemotherapy [11]. Collectively, no information about breast cancer molecular subtypes was presented in these studies.

Given the poor prognosis and aggressive characteristics of TNBC patients and the controversy surrounding PMRT as appropriate for patients with small tumors with 1–3 positive nodes, our study is designed to investigate the effect of postmastectomy radiotherapy on OS and breast cancer-specific survival (BCSS) among TNBC patients with T1-2 and 1-3 positive lymph nodes by utilizing population-based SEER data, and to further determine which patients are most likely to benefit from PMRT.

## RESULTS

### Demographic and clinical characteristics of the study population

A summary of demographic and clinical characteristics is shown in Table 1. A total of 675 patients met the eligibility criteria for our study, and all patients had undergone mastectomy. Among these patients, 53.8% (n=363) of the patients were classified as the no radiotherapy-received group (no-RT group), and 46.2% (n=312) as the radiotherapy-received group (RT group). The median follow-up time was 37 months. The median number of examined lymph nodes in our study was 13 for both groups. Compared with the patients who received RT, the no-RT group presented a significantly higher proportion of one positive lymph node (64.1% VS. 45.5%; P < 0.001). And 96.2% of the patients in RT group were offered chemotherapy while it was recorded that 78.8% of the no-RT group were offered chemotherapy. In addition, the other tumor characteristics, including age, race, marital status, laterality, histological grade and stage T status were similar between the two groups.

**Table 1:** Demographics and clinical characteristics of the study population

**Characteristics**	**No Radiotherapy (n=363)**		**Radiotherapy (n=312)**		**P^c^**
	**No.**	**%**	**No.**	**%**	
**Median follow-up (month) (IQR)**	37 (27-48)		37 (28-47)		
**Age (years)**					0.070
** Median**	53		50		
** <50**	147	40.5	148	47.4	
** ≥50**	216	59.5	164	52.6	
**Race**					0.225
** White**	275	75.8	219	70.2	
** Black**	64	17.6	64	20.5	
** Other^a^**	24	6.6	29	9.3	
**Marital status**					0.367
** Married**	231	63.6	188	60.3	
** Not married^b^**	132	36,4	124	39.7	
**Laterality**					0.413
** Left**	186	51.2	150	48.1	
** Right**	177	46.8	162	51.9	
**Histological Grade**					0.386
** I**	1	0.3	0	0.00	
** II**	45	12,4	31	9.9	
** III**^c^	317	87.3	281	90.1	
**Stage T**					0.468
** T1**	120	33.1	95	30.4	
** T2**	243	66.9	217	69.6	
**Number of lymph node**					**< 0.001**
** 1**	223	61.4	142	45.5	
** 2**	103	28.4	92	29.5	
** 3**	37	10.2	78	25.0	
**Chemotherapy**					**< 0.001**
** Yes**	286	78.8	300	96.2	
** No**	77	21.2	12	3.8	

Abbreviation: IQR, interquartile range.

^a^ Other includes American Indian/Alaskan native, and Asian/Pacific Islander.

^b^ Not married includes divorced, separated, single (never married), unmarried or domestic partner and widowed.

^c^ P value was calculated among all groups with a Chi-squared test, and a bold type indicates significance.

### Survival comparisons according to lymph node status and radiation status

**Figure 1:**
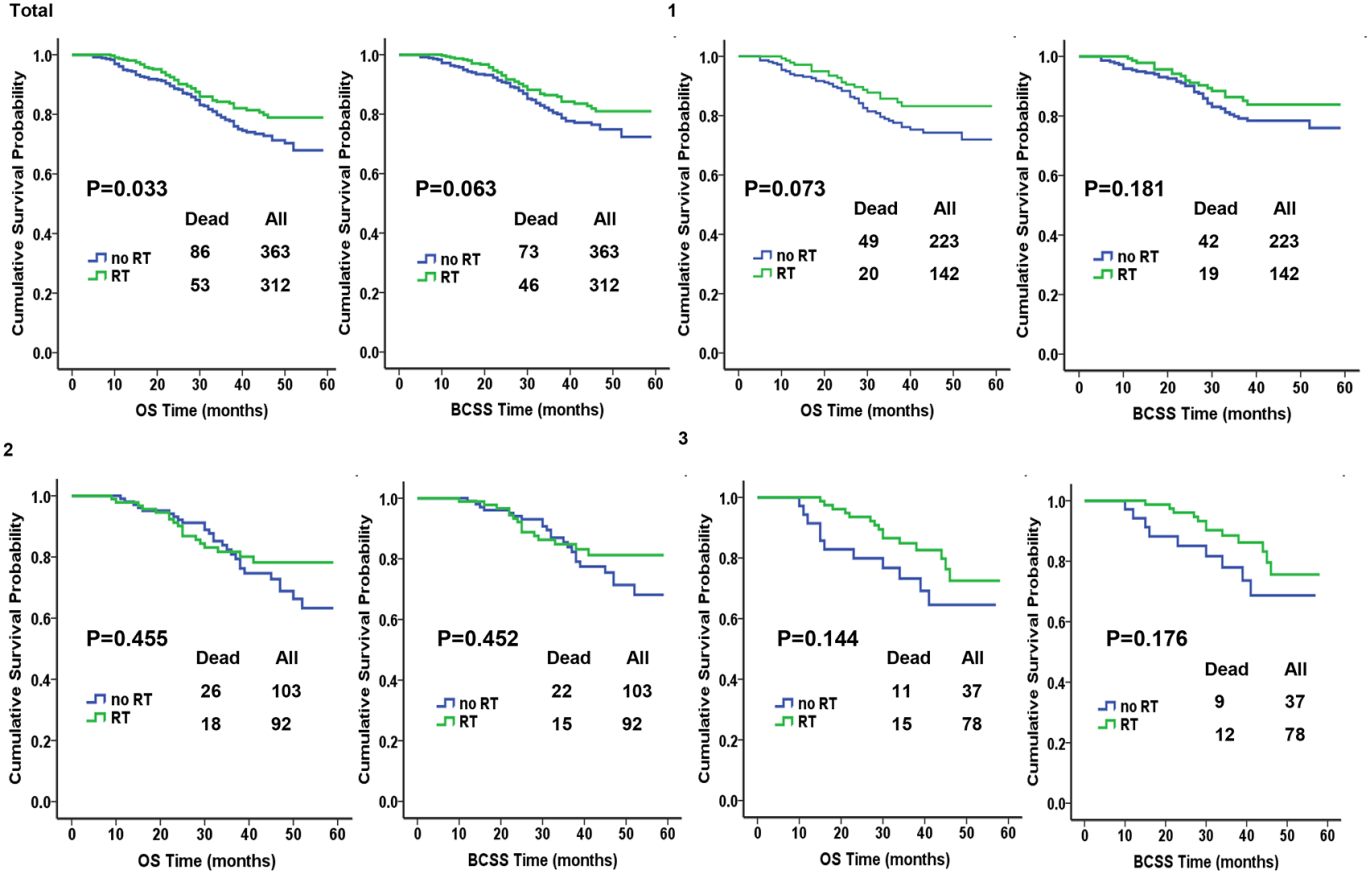
Kaplan-Meier plot and log-rank tests comparing overall survival (OS) and breast cancer-specific survival (BCSS) between RT and no-RT groups according to the number of positive lymph nodes.

We used the Kaplan-Meier plot and log-rank tests to compare BCSS and OS between different subgroups according to lymph node their status and the results are illustrated in Figure 1. In the total cohort of 675 patients, significantly improved OS after PMRT was found, whereas BCSS was similar between the RT and no-RT groups (*P*=0.033 and *P*=0.063). In addition, no significant differences in OS was observed between the groups stratified by the number of positive lymph nodes; *P*=0.073, *P*=0.455, *P*=0.144 for 1 LN, 2 LNs, and 3 LNs. there was no significant difference in BCSS when patients were stratified by the number of LN; *P*=0.181, *P*=0.452, and *P*=0.176 for 1 LN, 2 LNs and 3 LNs.

### Analyses of prognostic factors using Cox proportional hazard regression models

In order to further investigate the prognostic factors of patients with TNBC, BCSS and OS analyses via univariate and multivariate Cox proportional hazard regression models were performed and the results are presented in Supplementary Table 1 and Table 2, respectively. With univariate analyses, no married status was significantly associated with reduced OS and BCSS; hazard ratio (HR), 1.159 with a 95%CI, 1.117 to 2.174 and *P*=0.009 for OS and HR, 1.484 with a 95%CI, 1.035 to 2.127 and *P*=0.032 for BCSS. Compared with tumors sized 2 cm to 5 cm (T2), patients with ≤2 cm (T1) tumors had a better OS and BCSS with univariate analyses; HR, 0.617 with a 95%CI, 0.416 to 0.917 and *P*=0.017 for OS and HR, 0.548 with a 95%CI, 0.353 to 0.852 and *P*=0.008 for BCSS. This result retained significance in multivariate analyses of OS and BCSS (Table 2). In addition, PMRT was independently associated with increased OS according to univariate and multivariate analyses.

**Table 2:** Multivariate Cox proportional hazard model for the outcome-related factors

**Variable**	**OS**			**BCSS**		
	**HR**	**95%CI**	**P^a^**	**HR**	**95%CI**	**P^a^**
**Age (years)**						
** ≥50**	1			1		
** <50**	0.841	0.598-1.183	0.320	0.827	0.572-1.197	0.314
**Grade**						
** III**	1			1		
** I/II**	1.088	0.645-1.835	0.753	1.111	0.635-1.945	0.711
**Stage T**						
** T2**	1			1		
** T1**	0.607	0.408-0.902	**0.014**	0.544	0.349-0.846	**0.007**
**Number of lymph node**						
** 1**	1			1		
** 2**	1.121	0.768-1.638	0.553	1.065	0.707-1.603	0.764
** 3**	1.269	0.797-2.020	0.316	1.143	0.685-1.905	0.609
**Chemotherapy**						
** Yes**	1			1		
** No**	1.029	0.631-1.678	0.908	0.822	0.464-1.457	0.502
**Radiotherapy**						
** No**	1			1		
** Yes**	0.661	0.460-0.949	**0.025**	0.664	0.450-0.979	0.039

Abbreviation: CI, confidence interval.

^a^ P value was adjusted by a multivariate Cox proportional hazard regression model including age, grade, stage T, the number of positive lymph nodes, chemotherapy and radiotherapy, and a bold type indicates significance.

### Stratification analysis with the number of positive lymph nodes

In order to further investigate the effect of PMRT for TNBC patients, we performed multivariate analysis, stratifying according to the number of positive lymph node, which ruled out the impact of unbalanced distribution of chemotherapy between RT and no-RT groups. As shown in Table 3, when HRs of the three subgroups were conducted, no significant improvements in BCSS and OS were observed after PMRT.

**Table 3:** Multivariate Cox proportional hazard model assessing the effect of postmastectomy radiotherapy stratified by the number of positive lymph node

**Number of positive lymph node / Radiation**	**OS**			**BCSS**		
	**HR**	**95%CI**	**P^a^**	**HR**	**95%CI**	**P^a^**
**Total**						
** RT**	0.661	0.460-0.949	**0.025**	0.664	0.450-0.979	**0.039**
** no RT**	1			1		
**1 LN**						
** RT**	0.640	0.376-1.090	0.100	0.674	0.388-1.171	0.161
** no RT**	1			1		
**2 LNs**						
** RT**	0.765	0.415-1.411	0.391	0.741	0.381-1.441	0.377
** no RT**	1			1		
**3 LNs**						
** RT**	1			1		
** no RT**	1.841	0.795-4.267	0.155	1.796	0.693-4.656	0.228

Abbreviation: CI, confidence interval.

^a^ P value was adjusted by a multivariate Cox proportional hazard regression model including age, grade, stage T, chemotherapy and radiotherapy, and a bold type indicates significance.

## DISCUSSION

In the current study, we tried to investigate the effect of PMRT on BCSS and OS among TNBC patients with T1-2 and 1-3 positive lymph nodes utilizing population-based SEER data. We found an improved OS after PMRT in T1-2 and 1-3 axillary lymph node positive patients. This is the first study to show the difference of OS in such patients, while adjuvant radiotherapy has been reported to contribute to local-regional disease control in previous studies.

Currently, PMRT is recommended for patients with one to three nodes according to international consensuses and guidelines. The evidence comes mainly from a meta-analysis in 2014 from EBCTCG, which suggested PMRT could reduce 10-year rates of locoregional recurrence (LRR) and 20-year rates of breast cancer mortality [6]. But the treatment is still controversial because the trials from EBCTCG were predominantly performed in the 1970s and 1980s. In that period chemotherapy regime commonly was cyclophosphamide, methotrexate, and 5-fluorouracil (CMF) based, which is clearly inadequate for TNBC patients. The results do not reflect the advances in systemic therapy, as it has been confirmed that the taxane/anthracycline based chemotherapy regime could yield more significant benefits on survival than the CMF regimen [12–14]. In addition, the numbers of positive and dissected lymph nodes were considered as significant predictors for LRR [15], whereas the median number of resected lymph nodes was fewer than 10 in EBCTCG trial. Hence, this meta-analysis may not represent the current clinical practice. In contrast, our study recruited TNBC patients diagnosed from 2010 to 2012, who mainly received taxane/anthracycline-based chemotherapy regimens and the median number of examined lymph nodes was 13 for both of our study groups. Eliminating the above confounding factors, our results further forcibly corroborated that PMRT has an advantageous effect on the OS outcome for TNBC patients with T1-2 and 1-3 positive lymph nodes.

In clinical practice, considering the delayed complications from radiation including cardiac toxicity, lymphedema, skin fibrosis, and so on, some surgeons and radiation oncologist do not recommend PMRT to patients with 1-3 positive axillary lymph nodes. But TNBC is a special subtype of breast cancer. Patients with TNBC are likely to suffer poorer survival and are prone to early locoregional recurrence and distant metastasis. Previous studies have demonstrated that different molecular subtypes had different response to PMRT. Wen et al. [16] found that the HER2-enriched and TNBC subtypes were associated with significantly higher 5-year LRR rates, lower 5-year LRRFS rates, and poorer 5-year BCSS rates in pT1-2N1M0 breast cancer patients who did not undergo PMRT, compared with the luminal A subtype. This means that PMRT is more important in treatment of the TNBC than it is for luminal subtypes. But the effect of PMRT on TNBC patients remains controversial. Kyndi et al. [17] found that no significant OS improvement after PMRT was observed among patients with hormone receptor negative tumors. The authors speculated that this was due to the highly proliferative, aggressive behavior and radioresistance of TNBC subtype. Conversely, Chen et al. [18] confirmed the beneficial impact of PMRT on the DFS outcomes in patients with TNBC with intermediate-risk disease (stage T1–T2N1), which is consistent with our results. Accordingly, since TNBC is an aggressive disease and those patients can benefit from PMRT, why not strongly recommend them to receive PMRT to prolong survival? When making the decision to undergo PMRT or not, we should balance the benefits vs. toxicity and the tumor phenotype should be taken into consideration.

Although no statistical difference was observed for BCSS among the total cohort and by stratified analysis, a benefit trend was reflected from Kaplan-Meier analysis which was consistent with the trend in OS, thus these observations should be interpreted with caution. We speculated that the small percentage of patients who have died from breast cancer and the small sample size of patients who died in subgroup analysis could be one potential explanation for the finite benefits from PMRT. And hopefully will extending the follow-up time make our results more satisfactory.

There are several limitations of our study. Firstly, we were compelled to focus on short follow-up time because the information regarding HER-2 expression in the SEER database was not available until 2010. But for the TNBC subtype, an early peak of recurrence occurs within the first 2-3 years after diagnosis. Secondly, the SEER database is absent from information about LRR. However, information about chemotherapy is offered by the latest version of the SEER database. And we endeavored to mitigate several confounding variables, such as endocrine and trastuzumab treatment by selecting TNBC as the object of our study rather than other subtypes.

In conclusion, our study shares some similar results with the recent literature that there is a significant improvement on OS after PMRT among TNBC patients with T1-2 and 1-3 positive axillary lymph nodes. In accordance with current consensuses and guidelines, we suggest that PMRT should be strongly considered in T1-2N1 breast cancer patients particularly in TNBC, an aggressive and poor prognosis disease. Additional prospective randomized studies are needed to provide a stronger evidence base for selecting patients for PMRT.

## MATERIALS AND METHODS

### Ethics statement

The data in the SEER database do not require informed patient consent because cancer is a disease reported by every state of the United States. But a Data-Use Agreement for the SEER 1973–2014 Research Data File has been completed.

### Patients

SEER*Stat version 8.3.4 was used to generate a case listing. We identified 675 patients screened according to the following inclusion criteria: female; year of diagnosis from 2010 to 2012; age of diagnosis between 20 years and 80 years; breast cancer as the first and only malignant cancer diagnosis; pathologically confirmed invasive ductal carcinoma-not otherwise specified (ICD-O-3 8500/3), unilateral cancer; TNBC subtype (absence of ER, PR, and HER2); histological grades I-III; AJCC T stagesI-II; known tumor size category; known lymph node status; record of chemotherapy therapy, mastectomy received. We excluded patients with inflammatory breast cancer, *in situ* disease, and no record of radiotherapy. We calculated follow-up durations from January 1, 2010 to December 31, 2014 to ensure adequate follow-up duration.

### Statistical analyses

Patients were divided into RT group and no-RT group categorized by radiotherapy status. A Chi-squared test was used to compare the demographic and clinical characteristics of the included cases between the two groups. The Kaplan-Meier analysis was performed to generate survival curves, and the log-rank test was used to compare the unadjusted BCSS and OS rates of patients with different lymph node status. BCSS was measured from the date of diagnosis to the date of breast cancer death. OS was defined as the time from the date of diagnosis to the date of death due to any cause (including breast cancer) or the last follow-up. Adjusted hazard ratios (HRs) with 95% confidence interval (CI) were calculated using a Cox proportional hazard regression model to estimate the outcome-related factors. All *P*-values were two-sided and *P*<0.05 were considered statistically significant. All statistical analyses were carried out using SPSS version 22.0 software (IBM SPSS Statistics, Chicago, IL, US).

## SUPPLEMENTARY MATERIALS


