# Supplementary material for: Effect of postmastectomy radiotherapy on triple-negative breast cancer with T1-2 and 1-3 positive axillary lymph nodes: a population-based study using the SEER 18 database
Source: Oncotarget. 2019 Aug 27;10(50):5245–52. doi: 10.18632/oncotarget.24703 (PMC6718267; doi:10.18632/oncotarget.24703)
Supplement: Supplementary file 1 [file oncotarget-10-5245-s001.pdf]

Effect of postmastectomy radiotherapy on triple-negative breast cancer with T1-2 and 1-3 positive axillary lymph nodes: a population-based study using the SEER 18 database

SUPPLEMENTARY MATERIALS

Supplementary Table 1: Univariate Cox proportional hazard model for the outcome-related factors

Variable	OS			BCSS		
	HR	95%CI	P[c]	HR	95%CI	P[c]
**Age (years)**						
**≥50**	1			1		
**<50**	0.822	0.585-1.155	0.259	0.818	0.567-1.182	0.285
**Race**						
**White**	1			1		
**Black**	1.226	0.823-1.824	0.316	1.216	0.789-1.874	0.376
**Other[a]**	0.578	0.253-1.316	0.191	0.681	0.297-1.557	0.362
**Marital status**						
**Married**	1			1		
**Not married[b]**	1.559	1.117-2.174	**0.009**	1.484	1.035-2.127	**0.032**
**Grade**						
**III**	1			1		
**I/II**	1.077	0.640-1.814	0.779	1.108	0.634-1.936	0.718
**Stage T**						
**T2**	1			1		
**T1**	0.617	0.416-0.917	**0.017**	0.548	0.353-0.852	**0.008**
**Number of lymph node**						
**1**	1			1		
**2**	1.095	0.750-1.598	0.639	1.040	0.691-1.565	0.851
**3**	1.138	0.725-1.787	0.575	1.037	0.632-1.704	0.884
**Chemotherapy**						
**Yes**	1			1		
**No**	1.136	0.707-1.825	0.597	0.902	0.516-1.575	0.717
**Radiotherapy**						
**No**	1			1		
**Yes**	0.692	0.491-0.974	**0.035**	0.707	0.489-1.022	0.066

Abbreviation: CI, confidence interval.
[a] Other includes American Indian/Alaskan native, and Asian/Pacific Islander.
[b] Not married includes divorced, separated, single (never married), unmarried or domestic partner and widowed.
[c] P value was calculated by a univariate Cox proportional hazard regression model including all factors, and a bold type indicates significance.
